# Pathological Response in the Breast and Axillary Lymph Nodes after Neoadjuvant Systemic Treatment in Patients with Initially Node-Positive Breast Cancer Correlates with Disease Free Survival: An Exploratory Analysis of the GeparOcto Trial

**DOI:** 10.3390/cancers14030521

**Published:** 2022-01-20

**Authors:** Bernd Gerber, Andreas Schneeweiss, Volker Möbus, Michael Golatta, Hans Tesch, David Krug, Claus Hanusch, Carsten Denkert, Kristina Lübbe, Jörg Heil, Jens Huober, Beyhan Ataseven, Peter Klare, Markus Hahn, Michael Untch, Karin Kast, Christian Jackisch, Jörg Thomalla, Fenja Seither, Jens-Uwe Blohmer, Kerstin Rhiem, Peter A. Fasching, Valentina Nekljudova, Sibylle Loibl, Thorsten Kühn

**Affiliations:** 1 Department of Obstetrics and Gynecology, University of Rostock, Südring 81, 18059 Rostock, Germany; Bernd.Gerber@kliniksued-rostock.de; 2 National Center for Tumor Diseases, Heidelberg University Hospital and German Cancer Research Center, Im Neuenheimer Feld 460, 69120 Heidelberg, Germany; andreas.schneeweiss@med.uni-heidelberg.de; 3 Medical Clinic II, University Hospital Frankfurt, Theodor-Stern-Kai 7, 60590 Frankfurt, Germany; MoebusVolk@aol.com; 4 Department of Gynecology and Obstetrics, University of Heidelberg, Im Neuenheimer Feld 440, 69120 Heidelberg, Germany; michael.golatta@med.uni-heidelberg.de (M.G.); Joerg.Heil@med.uni-heidelberg.de (J.H.); 5 Oncology Practice, Bethanien Hospital Frankfurt, Im Prüfling 17-19, 60389 Frankfurt, Germany; hans.tesch@chop-studien.de; 6 Department of Radiotherapy, University Hospital Schleswig Holstein, Arnold-Heller-Straße 3, 24105 Kiel, Germany; david.krug@uksh.de; 7 Department of Senology, Rotkreuz-Klinikum, Rotkreuzplatz 8, 80634 Munich, Germany; claus.hanusch@swmbrk.de; 8 Institute of Pathology, Philipps-University Marburg, Baldingerstraße, 35043 Marburg, Germany; carsten.denkert@uni-marburg.de; 9 Breast Center, Diakovere Henriettenstift, Schwemannstraße 17, 30559 Hannover, Germany; Kristina.Luebbe@diakovere.de; 10 Department of Gynecology and Obstetrics, Ulm University Hospital, Albert-Einstein-Allee 23, 89081 Ulm, Germany; Jens.Huober@kssg.ch; 11 Department of Obstetrics and Gynecology, University Hospital, Ludwig Maximilian University of Munich, 81377 Munich, Germany; B.Ataseven@kem-med.com; 12 Department of Gynecology and Gynecologic Oncology, Kliniken Essen-Mitte, Henricistraße 92, 45136 Essen, Germany; 13 Oncologic Medical Care Center Krebsheilkunde, Möllendorffstraße 52, 10367 Berlin, Germany; info@drklare.de; 14 Department for Women’s Health, University of Tübingen, Calwerstraße 7, 72076 Tuebingen, Germany; Markus.Hahn@med.uni-tuebingen.de; 15 Department of Obstetrics and Gynecology, Helios Klinikum Berlin-Buch, Schwanebecker Chaussee 50, 13125 Berlin, Germany; michael.untch@helios-gesundheit.de; 16 Center for Hereditary Breast and Ovarian Cancer, University Hospital of Cologne, Kerpener Straße 62, 50937 Cologne, Germany; Karin.Kast@uniklinikum-dresden.de; 17 Department of Obstetrics and Gynecology, Sana Klinikum Offenbach GmbH, Starkenburgring 66, 63069 Offenbach, Germany; Christian.Jackisch@Sana.de; 18 Praxisklinik für Hämatologie und Onkologie Koblenz, Neversstraße 5, 56068 Koblenz, Germany; thomalla@onkologie-koblenz.de; 19 German Breast Group, Martin Behaim Strasse 12, 63263 Neu-Isenburg, Germany; fenja.seither@mailbox.org (F.S.); valentina.nekljudova@gbg.de (V.N.); 20 Department of Gynecology with Breast Center Charité, Charitéplatz 1, 10117 Berlin, Germany; jens.blohmer@charite.de; 21 Center for Hereditary Breast and Ovarian Cancer, Center for Integrated Oncology (CIO), Medical Faculty, University Hospital Cologne, Kerpener Straße 62, 50937 Cologne, Germany; kerstin.rhiem@uk-koeln.de; 22 Department of Obstetrics and Gynecology, University of Erlangen, Universitätsstraße 21/23, 91054 Erlangen, Germany; Peter.Fasching@uk-erlangen.de; 23 Department of Gynecology, Klinikum Esslingen, Hirschlandstraße 97, 73730 Esslingen, Germany; kuehn.thorsten@t-online.de

**Keywords:** breast cancer, neoadjuvant therapy, axillary surgery, pathological complete response, lymph node, prognosis

## Abstract

**Simple Summary:**

The extent of axillary surgery has been reduced in recent years to minimize side effects. However, a negative impact of reduced surgery on outcome must be avoided. We investigated for whom the extent of surgery can be safely reduced by examining early-stage breast cancer patients converting from lymph node (LN)-positive to LN-negative disease after neoadjuvant systemic treatment (NAST). Of 242 initially LN-positive patients treated within the GeparOcto trial, 54.5% were classified as LN-negative after NAST, 31.8% as LN-positive, and for 13.6% data were missing. Overall, 92.1% of patients underwent complete axillary LN dissection, with 6.6% undergoing sentinel LN dissection only. At surgery, 55.4% of patients had no signs of cancer in the LN, 45.0% had no signs of cancer in the breast (of those 8.3% had involved LN), and 41.3% had no signs of cancer at all. Patients with involved LN still had a bad prognosis. Conversion from LN-positive to LN-negative after NAST is of highest prognostic value. Surgical axillary staging after NAST is essential in these patients to offer tailored treatment.

**Abstract:**

Background: The conversion of initially histologically confirmed axillary lymph node-positive (pN+) to ypN0 after neoadjuvant systemic treatment (NAST) is an important prognostic factor in breast cancer (BC) patients and may influence surgical de-escalation strategies. We aimed to determine pCR rates in lymph nodes (pCR-LN), the breast (pCR-B), and both (tpCR) in women who present with pN+ BC, to assess predictors for response and the impact of pCR-LN, pCR-B, and tpCR on invasive disease-free survival (iDFS). Methods: Retrospective, exploratory analysis of 242 patients with pN+ at diagnosis from the multicentric, randomized GeparOcto trial. Results: Of 242 patients with initially pN+ disease, 134 (55.4%) had a pCR-LN, and 109 (45.0%) a pCR-B. Of the 109 pCR-B patients, 9 (8.3%) patients had involved LN, and 100 (41.3%) patients had tpCR. Those with involved LN still had a bad prognosis. As expected, pCR-B and intrinsic subtypes (TNBC and HER2+) were identified as independent predictors of pCR-LN. pCR-LN (ypN0; hazard ratio 0.42; 95%, CI 0.23–0.75; *p* = 0.0028 for iDFS) was the strongest independent prognostic factor. Conclusions: In initially pN+ patients undergoing NAST, the conversion to ypN0 is of high prognostic value. Surgical axillary staging after NAST is still essential in these patients to offer tailored treatment.

## 1. Introduction

The extent of axillary surgery is constantly decreasing in breast cancer surgery [1]. Since adjuvant decision making is increasingly based on biological and molecular factors, clinical trials are ongoing to investigate whether axillary staging (sentinel lymph node biopsy, SLNB) can be avoided completely in patients with early breast cancer and clinically unsuspicious nodes who undergo primary surgery [2].

Neoadjuvant systemic treatment (NAST) is becoming increasingly popular to de-escalate the surgical extent and provide prognostic information to tailor systemic treatment after surgery, especially in triple-negative (TNBC) and HER2-positive breast cancer [3,4].

Meta-analyses have shown that pathological complete response (pCR) after NAST is related to outcome and prognosis [5,6,7,8]. There are no data regarding pCR in breast and initially histologically proven metastatic involved lymph nodes and its impact on outcome.

The management of axillary lymph nodes in the context of NAST has been discussed intensively in recent years [9,10]. While SLNB has been accepted as an axillary staging procedure after NAST for cN0 patients, the surgical approach in patients who convert from histologically confirmed lymph node involvement (pN+) before NAST to clinically unsuspicious lymph nodes (ycN0) thereafter remains a matter of debate [11,12]. SLNB as a minimal invasive staging procedure is associated with a false-negative rate (FNR) of >14% in this group of patients, which is considered unacceptable by most surgeons [13]. New procedures that include the use of markers to locate a biopsy-proven positive lymph node and its targeted removal after NAST along with the SLN (targeted axillary dissection, TAD) were introduced recently and have shown false-negative rates consistently below 10% [12]. The risk for missing positive lymph nodes is, however, not only related to the FNR but also to risk of nodal involvement. The higher the pCR rates in node-positive patients, the lower the individual failure risk. Thus far, it is unclear if the FNRs associated with different staging procedures translate into oncologic outcome.

GeparOcto was a randomized phase III trial (NCT02125344) in high-risk early breast cancer that has shown similar pCR rates following NAST with intense dose-dense EPC or weekly PM(Cb) as previously published [14].

Here, we investigate the pCR rates in axillary lymph nodes (pCR-LN), breast (pCR-B), and both (tPCR) in patients with biopsy-proven initial axillary lymph node involvement (pN+). In addition, we evaluate predictors of pCR and assess the relation between pCR-LN, pCR-B, and tpCR and their relevance on outcome. The impact of these results on a risk-adapted surgical strategy for axillary management is discussed.

## 2. Materials and Methods

### 2.1. Study Design and Participants

GeparOcto was a neoadjuvant, randomized trial for patients with previously untreated, high-risk early breast cancer with an indication for chemotherapy [14]. From December 2014 through June 2016, 945 patients with high-risk early breast cancer were included and started treatment in 57 centers in Germany.

High-risk breast cancer was defined as TNBC or HER2-positive, independent of lymph node status or node-positive, luminal HER2-negative disease. Diagnosis of breast cancer was histologically confirmed by core biopsy. HER2-positive was defined as immunohistochemistry (IHC) 3+ or in situ hybridization (ISH) according to ASCO-CAP guidelines of 2013. HER2 and hormone receptor expression (negative if estrogen and progesterone receptor <1% by IHC) was assessed by central pathology. Lymphocyte-predominant breast cancer (LPBC) was defined as tumors having high tumor-infiltrating lymphocyte (TIL) levels (≥60%). All patients underwent disease staging by examination (breast and axilla), mammography, and ultrasound (breast und axillary lymph nodes). Distant metastases were excluded before study inclusion. Histologic confirmation of clinically suspicious (palpation and/or sonography) lymph nodes was highly recommended. Clipping of axillary lymph nodes was left to the discretion of the physicians.

The study was approved by the institutional review board and authorities and patients provided written informed consent. This study followed the Consolidated Standards of Reporting Trials (CONSORT) reporting guideline.

Patients were randomized in a 1:1 ratio to two different dose-intensified, dose-dense approaches: iddEPC (epirubicin 150 mg/m^2^ every 2 weeks for 3 cycles, followed by paclitaxel 225 mg/m^2^ every 2 weeks for 3 cycles, followed by cyclophosphamide 2000 mg/m^2^ every 2 weeks for 3 cycles) or PM(Cb) (paclitaxel 80 mg/m^2^ 18 times weekly, administered concurrent with NPLD (Myocet^®^ Transpharm Logistic GmbH, (Distributor on behalf of TEVA), Einstein Str. 2, 89179 Baimerstetten, Germany) 20 mg/m^2^ 18 times weekly, and in TNBC, administered concurrently with weekly carboplatin (AUC 1.5) 18 times). Dual HER2-blockade with trastuzumab and pertuzumab was given in HER2-positive disease.

### 2.2. Objectives

The primary aim of this analysis was the assessment of the pCR rate of axillary lymph nodes, of the primary tumor in the breast, and both after NAST in patients with histologically confirmed metastatic involved axillary lymph nodes at initial presentation. The second aim was to evaluate predictors for pCR-LN, pCR-B, and tpCR. As a third objective, we compared the impact of pCR rates in the 3 groups on invasive disease-free survival (iDFS). The overall objective was a risk assessment for surgical de-escalation strategies after NAST in pN+/ycN0 patients.

### 2.3. Assessment of Endpoints

After completion of NAST, all patients underwent standard breast surgery. The axillary approach was left to the discretion of the surgeons and to patient preference. After NAST, axillary lymph nodes were evaluated clinically (palpation and/or ultrasound) as ycN0, ycN+, or ycNx (missing data). Ultrasound of axillary lymph nodes was not recommended due to its low sensitivity. pCR was defined as no invasive residuals in the lymph nodes (ypN0), the breast (ypT0/is), or both tpCR (ypT0/is ypN0) after NAST. All removed axillary lymph nodes were examined by hematoxylin-eosin staining only. IHC of lymph nodes for cytokeratin was not performed. Micrometastases (ypN1mi) were defined as ypN1. All histopathological reports were centrally reviewed for pCR assessment. Invasive disease-free survival was defined as no ipsilateral regional invasive breast cancer recurrence, distant recurrence, contralateral invasive breast cancer, or death.

### 2.4. Statistical Analysis

Cross-tables with 2-sided Fisher’s exact test (for binary parameters) or χ^2^-test were used to report and compare rates. Median and range was reported for continuous parameters. Multivariate logistic regressions were performed for different pCR definitions to compare rates according to histological nodal status at baseline. Those were adjusted for other baseline parameters and treatment arm and odds ratios (OR) and their 95% confidence intervals (CI) were reported. For iDFS, Kaplan–Meier curves were plotted and compared with log-rank test; Cox proportional hazard model was used to report hazard ratio (HR) with 95% CI. All statistical analyses were performed with the use of SAS software, version 9.4.

## 3. Results

### 3.1. Patient and Tumor Baseline Characteristics

In total, 945 patients with a high-risk early breast cancer were included in the GeparOcto trial and started treatment. The majority of the patients presented with TNBC (*N* = 403, 42.6%) or HER2-positive disease (*N* = 382, 40.4%). Four hundred and thirty-one (46.2%) of the 945 patients had clinically suspicious or positive lymph nodes at the time of presentation. Initially nodal involvement by core biopsy (pN+) was confirmed in 242 (25.6%) patients (Figure S1). The baseline characteristics of these 242 patients before NAST are shown in Table S1. The median age was 47 years (range 21–74) and the median tumor size assessed by ultrasound was 27 mm (range 8–147). Sixty-three patients (26.0%) had TNBC, while 69 women (28.5%) presented with HER2-positive and 110 (45.5%) with luminal HER2-negative disease.

### 3.2. Management of Axillary Lymph Nodes after Neoadjuvant Systemic Treatment

After NAST, 132 of 242 (54.5%) patients were staged as ycN0, 77 (31.8%) as ycN+, and 33 (13.6%) as ycNx (missing data). Overall, 223 (92.1%) patients (ycN0: 94.6%, ycN+: 90.9%, and ycNx: 93.9%) underwent complete ALND. A regular SLNB with single tracer alone was performed in 16 (6.6%) of 242 patients (Table S2).

### 3.3. Pathological Response after NAST

Seventy-three out of 132 patients were staged as ycN0 (55.4%) and had a pCR-LN, while in ycN+ patients, the ypN0 rate was as low as 57.1% and for ypNx 51.5%. An overall pCR-LN was observed in 134/242 (55.4%) of initially pN+ patients. Micrometastases were found in eight patients with ypN1mi and two with ypN0 i+, respectively. The ypN0 rate was highly related to pCR-B (92.0%) and tumor subtype (81.6% for HER2-positive, 75.0% for TNBC, and 25.9% for luminal HER2-negative disease) (Table 1). A pCR-B irrespective of nodal status was confirmed in 109 (45.0%) patients with significant differences between subtypes (71.0% in HER2-positive, 65.1% in TNBC, and 17.3% in luminal HER2-negative). A tpCR was observed in 100 patients (41.3%), with comparable rates in HER2-positive (66.7%) and TNBC (60.3%) and a significantly lower rate in luminal HER2-negative (14.6%; *p* < 0.001) patients (Table S3).

Multivariate logistic regression analysis revealed pCR-B as the strongest predictor for pCR-LN (OR 31.7, 95% CI 12.0–84.0). Pre-NAST tumor stage (cT4 vs. cT1-3, OR 0.15, 95% CI 0.03–0.63; *p* = 0.01), grading (G3 vs. G1–2, OR 2.19, 95% CI 1.12–4.28), tumor biology (HER2-positive vs. luminal HER2-negative, OR 8.94, 95% CI 4.02–19.89; *p* < 0.001; TNBC vs. luminal HER2-negative, OR 3.55, 95% CI 1.71–7.37, *p* < 0.001), and LPBC (yes vs. no, OR 3.39, 95% CI 1.29–8.96, *p* = 0.014) were further identified as independent predictors of pCR-LN.

For pCR-B (ypT0/is) and tpCR only, the biological subtype (TNBC and HER2-positive versus luminal HER2-negative) and LPBC were identified as independent predictors. There were no significant pCR differences between both arms (Table S4).

### 3.4. Invasive Disease-Free Survival in Patients with Initially Lymph Node-Positive Breast Cancer

**Figure 1:**
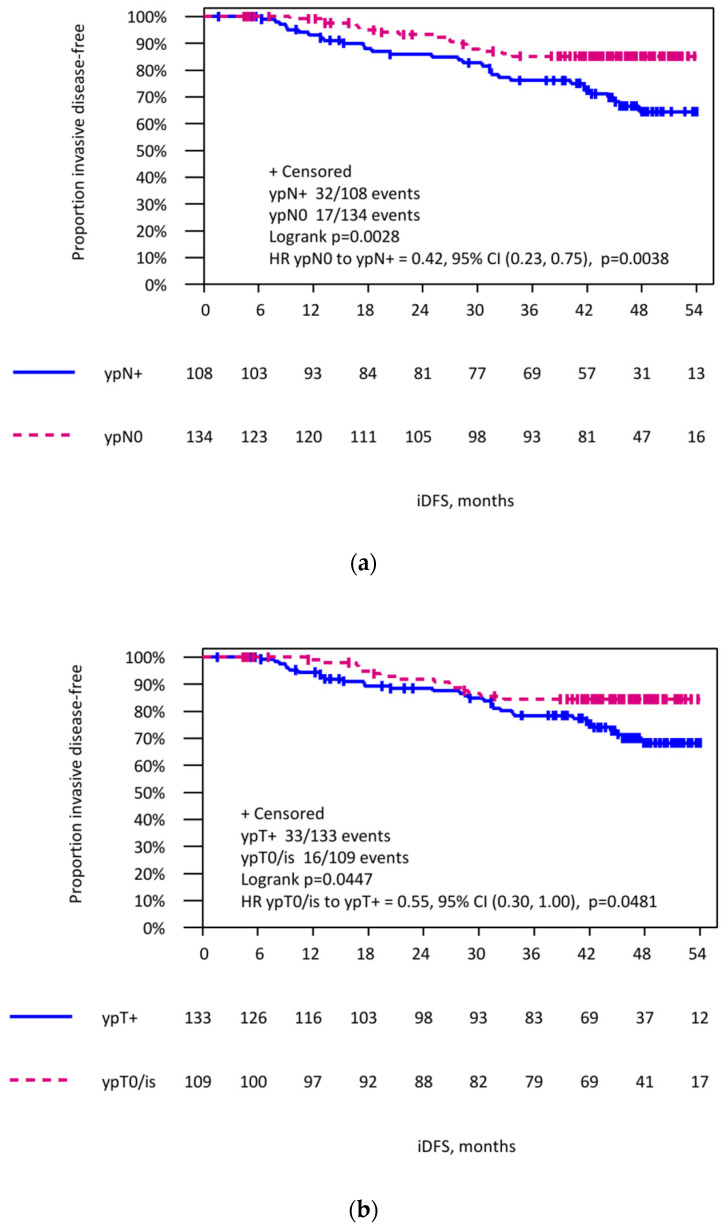

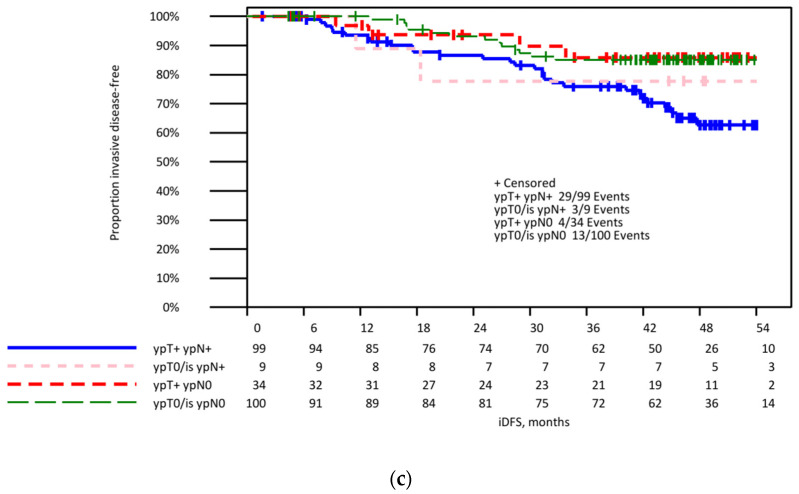
Invasive disease-free survival in patients with initially lymph node-positive breast cancer. (**a**) In patients with pcR (ypN0), respectively, non-pCR (ypN+) in lymph nodes after neoadjuvant systemic treatment. (**b**) According to the pCR in breast and nodes (ypT0/is ypN0), after neoadjuvant systemic treatment. (**c**) Invasive disease-free survival in patients with initially lymph node-positive breast cancer according to the pCR in breast (ypT0/is) and nodes (ypN0) after neoadjuvant systemic treatment.

After a median follow-up period of 46.5 months, patients with pCR-LN had a significantly better iDFS compared to patients with residual tumor in the lymph nodes (HR 0.42; 95% CI 0.23–0.75; log-rank *p* = 0.0028). A smaller difference in iDFS was observed between patients with a pCR-B alone compared to patients with a non-pCR-B (HR 0.55; 95% CI 0.30–1.00; log-rank *p* = 0.045). Irrespective of the tumor residuals in the breast, the persistent lymph node involvement was the strongest predictor for iDFS (Figure 1; ypT0/is ypN0 vs. ypT+ ypN+ HR 0.42, 95% CI 0.22–0.80, *p* = 0.009; ypT+ ypN0 vs. ypT+ ypN+ HR 0.40, 95% CI 0.14–1.14, *p* = 0.087; ypT0/is ypN+ vs. ypT+ ypN+ HR 0.88, 95% CI 0.27–2.93, *p* = 0.84).

## 4. Discussion

Surgical de-escalation strategies in lymph node surgery after NAST are currently intensively debated. Sentinel lymph node biopsy has replaced ALND as treatment in cN0 patients even after NAST. A retrospective analysis of the National Cancer Data Base (NCDB) in cN0 patients who presented with HER2-positive or TNBC disease and had a pCR-B demonstrated a rate of nodal involvement after NAST below 2%. The authors concluded that any axilla surgery might be omitted in this subset of patients [15]. In view of these data, new prospective trials are ongoing to omit any lymph node surgery in these women (EUBREAST1-trial, NCT04101851). In a pooled analysis with 57,531 unique patients, pCR-LN rates were reported in 60% of hormone receptor-negative/HER2-positive, 48% of TNBC, 45% of hormone receptor-positive/HER2-positive, 35% of luminal B, 18% of hormone receptor-positive/HER2-negative, and 13% of luminal A breast cancer. The pCR rates were independent of clinically or pathologically proven node status [16].

In the light of those data and initiatives, we investigated in the pN+ subset of the patients enrolled in the neoadjuvant GeparOcto study the rate of ypN+ patients in patients with a breast pCR and its prognostic value. The rates for pCR-LN, pCR-B, and tpCR were 55.4%, 45.0%, and 41.3%, respectively. This shows that at least 8% of these high-risk patients with a breast pCR still had nodal involvement. These data also reveal that more than 50% of patients converted to a histologically negative lymph node status and would be overtreated with full ALND. Minimal invasive axillary surgery to reliably assess the lymph node status after NAST appears therefore crucial. This result is comparable to data reported from a prospective cohort study presented by Tadros et al., which showed a ypN0 stage of almost 90% for patients with biopsy proven pN+, who had a pCR-B [17]. These data were confirmed by another retrospective analysis from the NCDB on 30,821 patients with a ypN+ rate of 12.4% in this cohort of patients [15]. In contrast, Samiei et al. demonstrated a nodal involvement rate of 45% after NAST for cN+ patients with pCR-B [18]. The difference of clinically nodal involvement and histologically proven nodal involvement is important and must be considered.

In addition, as expected, breast pCR was identified as the most important predictor of pCR-LN.

For patients with initially node-positive disease, the optimal staging procedure to assess the ypN status is currently not clearly defined. The SLNB technique is associated with an FNR of around 14%, which is considered as unacceptably high by most surgeons. New techniques such as the TAD, that combines SLNB with the removal of a (pre-NAST) biopsy-proven lymph node (target lymph node), that may be located by a multitude of different markers, is becoming increasingly popular due to an FNR lower than 7% [19], respectively <9% [20,21]. Barrio et al. recently reported that in patients with cN1 disease converted to ycN0 and three or more negative SLN after SLNB alone, and nodal recurrence rates were low, without routine nodal clipping [22]. These findings would also support omitting ALND in these patients. Thus far, however, no data are available that provide prospective evidence on the oncologic outcome of different surgical staging procedures and provide information and the associated quality of life. The prospective AXSANA study (EUBREAST-03, NCT4373685) addresses these issues [20].

The individual risk of missing tumor involvement with a minimal invasive staging procedure (with its associated specific FNR) depends on the rate of patients with a complete tumor response in the lymph nodes.

The prognostic significance of low-volume residual nodal disease after NAST (ypN0 i+/ypN1(mi)) is unclear. In an analysis of more than 36,000 patients from Dana-Farber/Brigham and Women’s Cancer Center (DFBWCC) and the NCDB, patients with ypN0(i+) and ypN1(mi) disease had a twofold higher risk of death compared to ypN0 patients (*p* < 0.001) [23]. In another series of 134 patients with initially node-positive disease, the incidence of breast cancer-related, loco-regional events and death from BC were similar between patients with unaffected SLNs and women with micrometastatic lymph node involvement (28.9% vs. 30.2%, *p* = 0.954; 21.6% vs. 13.4%, *p* = 0.840; 12.9% vs. 24.5%, *p* = 0.494). Overall survival and disease-free survival were lower for patients with macrometastatic disease in the SLN [24]. In 59% of patients with micrometastatic lymph node involvement after NACT, Moo et al. found one or more additional positive nodes at ALND. They concluded that in patients with low-volume SLN metastases after NAST completion ALND should be performed [25].

The ongoing ALLIANCE 11202 trial (NCT01901094) compared ALND with axillary radiation for patients with a positive LN after NAST. The results might reduce axillary surgery in this cohort.

Current diagnostic tools such as palpation, ultrasound, and even magnetic resonance imaging (MRI) for evaluation of the axillary lymph node status following NAST have an unacceptable low accuracy, even after inclusion of clinico-pathological criteria [25,26,27,28,29,30,31]. Therefore, in patients with initially affected axillary lymph nodes and ycN0 after NAST, an evaluation of lymph nodes by imaging alone cannot be recommended. Half of ycN0 patients still have histologically involved lymph nodes. Vice versa, we observed a pCR rate in 57.1% of the patients with clinically affected lymph nodes (ycN+) after NAST. This underscores the importance of assessing the histopathological axillary lymph node status after NAST.

In our study, we found a strong relation between tumor subtype and pCR-LN, pCR-B, and tpCR. While the pCR-LN rate was as high as 81.6% for HER2-positive and 75.0% for TNBC, it was only 25.9% in luminal HER2-negative tumors. For pCR-B, the rates were 71.0%, 65.1%, and 17.3%, respectively. A similar relation between response and tumor subtype was reported from Barron et al. with pCR-B rates of 43.3% in HER2-positive, 37.7% in TNBC, and 12.7% in luminal HER2-negative disease [15]. In a pooled analysis of 33 studies with 57,531 clinically node-positive patients before NAST axillary pCR according to subtypes were reported in 60% (hormone receptor-negative/HER2-positive), 59% (HER2-positive/hormone receptor-negative or positive), 48% TNBC, 45% (hormone receptor-positive/HER2-positive), 35% (luminal B), 18% (hormone receptor-positive/HER2-negative), and 13% (luminal A) breast cancer [16]. The difference in magnitude of the effect of NAST on pCR rates may be explained by a modern dose-dense intensified schedule as used in the GeparOcto trial. To our knowledge, this is the first study to analyze the effect of pCR-LN, pCR-B, and tpCR on iDFS in patients with initially pN+ separately. We found that pCR-LN, pCR-B, and tpCR are associated with improved iDFS. However, pCR-LN is the strongest predictor for iDFS. These data indicate that irrespective of tumor residuals in the breast, the persistent lymph node involvement drives the prognosis.

A strength of our multicenter trial is the initial histological confirmation of lymph node status by core biopsy in all patients with suspicious lymph nodes. Patients who received an SLNB for cN+ were excluded. Patients were treated in a prospectively randomized trial with a specific systemic treatment and data on axillary diagnostic and treatment were captured predefined. Most patients received axillary lymph node dissection according to the historical guidelines offering robust data on pCR-LN. A limitation is the unplanned and retrospective design of this analysis and the small patient number.

## 5. Conclusions

In conclusion, lymph node involvement is the major driver of prognosis after NAST. Surgical staging of the axilla in pN+ patients who convert to ycN0 remains important because 50% overall and 8.3% of the breast pCR patients still had involved lymph nodes. Patients with a good response in the breast have a low likelihood of residual axillary disease. Axillary dissection to stage the axilla should be avoided as an overtreatment. For patients who convert from pN+ to ycN0, minimal invasive staging procedures (SLNB, TAD) should be recommended. Prospective studies to define outcome and quality of life of different techniques are warranted.

## Figures and Tables

**Table 1:** Pathological lymph node status after NAST according to clinical nodal status after NAST and biological subtype.

	Biological Subtype	ypN0 N (%)	ypN+ N (%)	*p*-Value ^a^
ycN0	luminal HER2- (*N* = 58)	15 (25.9)	43 (74.1)	<0.001
	TNBC (*N* = 36)	27 (75.0)	9 (25.0)	
	HER2+ (*N* = 38)	31 (81.6)	7 (18.4)	
	Overall (*N* = 132)	73 (55.3)	59 (44.7)	
ycN+	luminal HER2- (*N* = 35)	14 (40.0)	21 (60.0)	0.010
	TNBC (*N* = 21)	13 (61.9)	8 (38.1)	
	HER2+ (*N* = 21)	17 (81.0)	4 (19.0)	
	Overall (*N* = 77)	44 (57.1)	33 (42.9)	
ycNx *	luminal HER2- (*N* = 17)	6 (35.3)	11 (64.7)	0.080
	TNBC (*N* = 6)	3 (50.0)	3 (50.0)	
	HER2+ (*N* = 10)	8 (80.0)	2 (20.0)	
	Overall (*N* = 33)	17 (51.5)	16 (48.5)	

Abbreviations: TNBC, triple-negative breast cancer; NAST, neoadjuvant systemic treatment; ^a^ comparing ypN0 rate between subtypes; * missing data.

## Data Availability

The data presented in this study are available on request for researchers who provide translational research proposals. Proposals should be directed to http://www.gbg.de/de/forschung/translationale-forschung.php, (accessed on 28 November 2021); to gain access, data requestors will need to sign a data transfer agreement.

## Supplementary Materials

The following supporting information can be downloaded at: https://www.mdpi.com/article/10.3390/cancers14030521/s1, Figure S1: Consort diagram, Table S1: Baseline characteristics of included patients before neoadjuvant systemic treatment, Table S2: Management of axillary lymph nodes after neoadjuvant systemic treatment dependent on clinical lymph node status, Table S3: pCR rates in lymph nodes and breast according to different definitions and according to biological tumor type in patients with pN+ at baseline, Table S4: Multivariate logistic regression for pCR-LN (with and without pCR-B as a covariate), pCR-B and for tpCR in all 242 patients with pN+ by biopsy.
