# Supplementary material for: Pathological Response in the Breast and Axillary Lymph Nodes after Neoadjuvant Systemic Treatment in Patients with Initially Node-Positive Breast Cancer Correlates with Disease Free Survival: An Exploratory Analysis of the GeparOcto Trial
Source: Cancers (Basel). 2022 Jan 20;14(3):521. doi: 10.3390/cancers14030521 (PMC8833390; doi:10.3390/cancers14030521)
Supplement: Supplementary file 1 [file cancers-14-00521-s001.zip › cancers-1513332-supplementary.pdf]

Figure S1. Consort diagram.

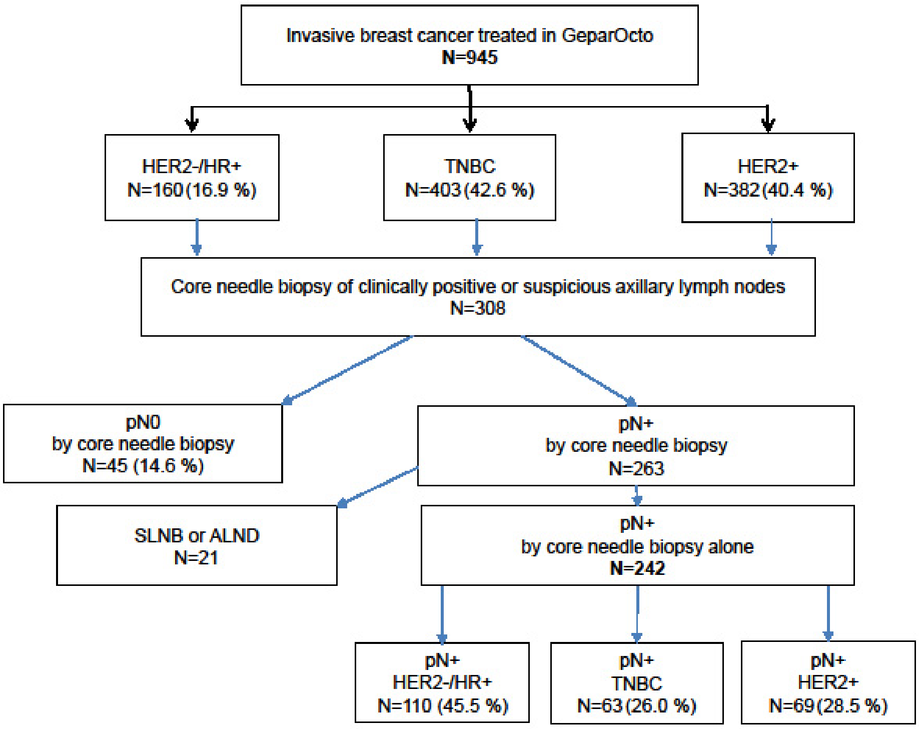

Table S1. Baseline characteristics of included patients before neoadjuvant systemic treatment.

Parameter	Category/ Statistic	pN+ biopsy (N=242) N (valid %)
Age, median (range), years	47 (21, 74)	
Age (at diagnosis), years	< 40	61 (25.2)
	≥ 40	181 (74.8)
Menopausal status	Premenopausal	150 (62.0)
	postmenopausal	92 (38.0)
BMI, kg/m²	< 25	122 (50.4)
	≥ 35	120 (49.6)
cT	cT1	69 (28.7)
	cT2	144 (60.0)
	cT3	11 (4.6)
	cT4	16 (6.7)
	missing	2
Tumor size by ultrasound, median (range), mm	27 (8, 147)	
	missing	2
Tumor localization	Unilateral	235 (97.1)
	Bilateral	7 (2.9)
Tumor focality	Unifocal	172 (71.1)
	Multifocal/centric	70 (28.9)
Grading	G1	2 (0.8)
	G2	75 (31.0)
	G3	165 (68.2)
Biological subtype	HR+ HER2-	110 (45.5)
	TNBC	63 (26.0)
	HER2+	69 (28.5)
Ki-67 (central)	≤20 %	10 (4.1)
	>20%	232 (95.9)
Histological tumor type	ductal or ductal-lobular	201 (83.1)
	other	41 (16.9)
LPBC	no	209 (86.4)
	yes	33 (13.6)
Systemic treatment	EPC	118 (48.8)
	PM(Cb)	124 (51.2)

Abbreviations: BMI, body-mass index; EPC, epirubicin, paclitaxel, cyclophosphamide; HR, hormone receptor; LPBC, lymphocyte-predominant breast cancer; PM(Cb), paclitaxel, non-pegylated liposomal doxorubicin (M, Myocet®), carboplatin; TNBC, triple-negative breast cancer

Table S2. Management of axillary lymph nodes after neoadjuvant systemic treatment dependent on clinical lymph node status.

ycN	Axilla surgery	pN+ biopsy (N=242) N (%)
ycN0	None	0 (0.0)
	SLNB	7 (5.4)
	ALND	122 (94.6)
	missing	3
	total	132 (54.5)
ycN+	None	0 (0.0)
	SLNB	7 (9.1)

ycN	Axilla surgery	pN+ biopsy (N=242) N (%)
	ALND	70 (90.9)
	missing	0
	total	77 (31.8)
ycNx[a]	None	0 (0.0)
	SLNB	2(6.1)
	ALND	31 (93.9)
	missing	0
	total	33 (13.6)

Abbreviations: ALND, axillary lymph node dissection; SLNB, sentinel lymph node biopsy. [a] ycNx = missing clinical axilla evaluation before surgery

Table S3. pCR rates in lymph nodes and breast according to different definitions and according to biological tumor type in patients with pN+ at baseline.

pCR definition	Biological subtype	pCR N (%)	No pCR N (%)	p-value[e]
ypT0/Tis ypN0[a]	HR+ HER2- (N=110)	16 (14.6)	94 (85.4)	<0.001
	TNBC (N=63)	38 (60.3)	25 (39.7)	
	HER2+ (N=69)	46 (66.7)	23 (33.3)	
	Overall (N=242)	100 (41.3)	142 (58.7)	
ypT0 ypN0[b]	HR+ HER2- (N=110)	14 (12.7)	96 (87.3)	<0.001
	TNBC (N=63)	36 (57.1)	27 (42.9)	
	HER2+ (N=69)	41 (59.4)	28 (40.6)	
	Overall (N=242)	91 (37.6)	151 (62.4)	
ypT0/Tis ypN0/+[c]	HR+ HER2- (N=110)	19 (17.3)	91 (82.7)	<0.001
	TNBC (N=63)	41 (65.1)	22 (34.9)	
	HER2+ (N=69)	49 (71.0)	20 (29.1)	
	Overall (N=242)	109 (45.0)	133 (55.0)	
ypT0 ypN0/+[d]	HR+ HER2- (N=110)	14 (12.7)	96 (87.3)	<0.001
	TNBC (N=63)	39 (61.9)	24 (38.1)	
	HER2+ (N=69)	42 (60.9)	27 (39.1)	
	Overall (N=242)	95 (39.3)	147 (60.7)	
ypN0	HR+ HER2- (N=110)	35 (31.8)	75 (68.2)	<.001
	TNBC (N=63)	43 (68.3)	20 (31.7)	
	HER2+ (N=69)	56 (81.2)	13 (18.8)	
	Overall (N=242)	134 (55.4)	108 (44.6)	

Abbreviations: HR, hormone receptor; pCR, pathological complete response; TNBC, triple-negative breast cancer;

[a] ypT0/Tis ypN0: no invasive residuals in breast and nodes; intraductal residuals allowed

[b] ypT0 ypN0: no invasive and no non-invasive residuals in breast and lymph nodes

[c] ypT0/Tis ypN0/+: no invasive residual tumor in the breast, intraductal residuals allowed, irrespective of nodal status after therapy

[d] ypT0/Tis ypN0/+: no invasive and no non-invasive residuals in the breast, irrespective of nodal status after therapy

[e] comparing pCR rates between biological subtypes for each definition

Table S4. Multivariate logistic regression for pCR-LN (with and without pCR-B as a covariate), pCR-B and for tpCR in all 242 patients with pN+ by biopsy.

		pCR-LN (ypN0)			pCR-LN (ypN0) with pCR-B in the model			pCR-B (ypT0/is ypN0/+)			tpCR (ypT0/is ypN0)		
**Parameter**	**Level**	**OR**	**95% CI**	**p-value**	**OR**	**95% CI**	**p-value**	**OR**	**95% CI**	**p-value**	**OR**	**95% CI**	**p-value**
Age, years	<40												
	≥40	0.87	(0.44, 1.74)	0.695	1.38	(0.57, 3.34)	0.474	0.51	(0.24, 1.05)	0.068	0.59	(0.28, 1.22)	0.153
cT	cT4												
	cT1-3	6.80	(1.58, 29.3)	0.010	14.6	(2.35, 90.3)	0.004	1.44	(0.37, 5.50)	0.598	3.33	(0.77, 14.4)	0.108
Tumor size by ultrasound, mm	≤23.0 (median)												
	>23.0	0.90	(0.49, 1.67)	0.738	1.06	(0.50, 2.36)	0.885	0.75	(0.40, 1.41)	0.375	1.00	(0.53, 1.88)	0.997
Biological subtype	HER2-/HR+			<.001			0.096			<.001			<.001
	TNBC	3.55	(1.71, 7.37)	<.001	1.04	(0.38, 2.80)	0.943	7.79	(3.59, 16.9)	<.001	7.48	(3.41, 16.41)	<.001
	HER2+	8.94	(4.02, 19.89)	<.001	2.69	(1.04, 6.96)	0.041	12.6	(5.77, 27.7)	<.001	12.21	(5.52, 27.01)	<.001
Grading	G1-2												
	G3	2.19	(1.12, 4.28)	0.022	2.59	(1.13, 5.96)	0.025	1.29	(0.64, 2.61)	0.483	1.59	(0.78, 3.27)	0.205
Ki67, %	<20%												
	≥20%	0.89	(0.16, 4.92)	0.891	0.77	(0.10, 5.97)	0.800	1.15	(0.25, 5.31)	0.858	0.89	(0.19, 4.10)	0.878
LPBC	no												
	yes	3.39	(1.29, 8.96)	0.014	2.17	(0.66, 7.13)	0.202	3.08	(1.24, 7.68)	0.016	2.69	(1.10, 6.53)	0.030
Histological tumor type	ductal or ductal-lobular invasive												

		pCR-LN (ypN0)			pCR-LN (ypN0) with pCR-B in the model			pCR-B (ypT0/is ypN0/+)			tpCR (ypT0/is ypN0)		
**Parameter**	**Level**	**OR**	**95% CI**	**p-value**	**OR**	**95% CI**	**p-value**	**OR**	**95% CI**	**p-value**	**OR**	**95% CI**	**p-value**
	other	1.03	(0.46, 2.31)	0.942	0.88	(0.31, 2.51)	0.817	1.32	(0.59, 2.98)	0.499	1.17	(0.52, 2.65)	0.702
Arm	EPC												
	PM(Cb)	0.89	(0.49, 1.64)	0.713	0.67	(0.32, 1.41)	0.292	1.28	(0.68, 2.38)	0.445	1.41	(0.75, 2.65)	0.285
pCR-B	ypT+	-	-	-				-	-	-	-	-	-
	ypT0/is	-	-	-	31.7	(12.0, 84.0)	<.001	-	-	-	-	-	-

Abbreviations: CI, confidence interval; EPC, epirubicin, paclitaxel, cyclophosphamide; HR, hormone receptor; LPBC, lymphocyte-predominant breast cancer; OR, odds ratio; PM(Cb), paclitaxel, non-pegylated liposomal doxorubicin (M, Myocet®), carboplatin; TNBC, triple-negative breast cancer;
